# Maternity care bundle for UK women with multiple long-term health conditions: coproduction workshops

**DOI:** 10.1136/bmjopen-2025-103366

**Published:** 2026-02-06

**Authors:** Stephanie Hanley, Sharon McCann, Megha Singh, Zoe Vowles, Siang Ing Lee, Rachel Plachcinski, Krishnarajah Nirantharakumar, Mairead Black, Louise Locock, Beck Taylor

**Affiliations:** 1 Department of Applied Health Sciences, University of Birmingham, Birmingham, UK; 2 Aberdeen Centre for Evaluation, University of Aberdeen, Aberdeen, UK; 3 Guy’s and St. Thomas’ NHS Foundation Trust, London, UK; 4 Aberdeen Centre for Women’s Health Research, Aberdeen Maternity Hospital, Aberdeen, UK; 5 Department of Health Sciences, University of Warwick, Coventry, UK

**Keywords:** Pregnancy, Postpartum Period, Delivery of Health Care, Integrated, QUALITATIVE RESEARCH

## Abstract

**Abstract:**

**Objective:**

The objective of this study is to co-produce a care bundle for women with multiple long-term health conditions (MLTC) that could be pilot tested and implemented in UK maternity services.

**Design:**

Online co-production workshops each attended by 20–30 key interest holders.

**Setting:**

United Kingdom, October 2023-February 2024.

**Population:**

Women with experience of pregnancy with MLTC, healthcare professionals and other interest holders involved in commissioning, planning and delivering care for pregnant women with MLTC.

**Methods:**

This study followed a three-step process: (1) a consolidated list of key components of care for pregnant women with MLTC was created through secondary analysis of prior collected qualitative data; (2) the list of care components was explored during four co-production workshops; and (3) findings from (1) and (2) were synthesised to develop a maternity care bundle of 4–5 key care components for pregnant women with MLTC.

**Main outcome measures:**

A maternity care bundle of five key care components for pregnant women with MLTC.

**Results:**

A list of 25 care components was refined to develop a proposed care bundle of five components. These were provisions of early and reliable medication advice and decision support; creation of a ‘goals of care summary’ accessible to women and the care team; provision of continuity of midwifery care throughout pregnancy and postnatal care; provision of a named care coordinator; and a formal postnatal handover of care from the multidisciplinary care team to the General Practitioner (GP) and secondary care team involving the woman.

**Conclusions:**

This study coproduced an evidence-based care bundle for pregnant women with MLTC to enhance communication and ensure individualised care and support. Further collaborative work with women and professionals is required to refine, implement and evaluate its impact on outcomes.

**STRENGTHS AND LIMITATIONS OF THIS STUDY**

- This is the first study to explore the development of a care bundle for pregnant women with multiple long-term conditions, spanning the full pregnancy pathway from preconception to postnatal care.
- The study is grounded in extensive qualitative research, ensuring the care components are informed by women’s lived experiences and clinical perspectives.
- Co-production workshops enabled collaboration among diverse stakeholders, including women, healthcare professionals, researchers and policymakers.
- Systematic evaluation of care components considered their priority, feasibility and potential impact on outcomes.
- The virtual workshop format facilitated national participation but may have limited interaction quality and representation of some clinical specialties.

## Introduction

 At least one in five women enter pregnancy with multiple long-term health conditions (MLTC),^1^ which is linked to increased risk of adverse health outcomes for mothers and babies.^2 3^ The majority of UK maternal deaths involve co-occurring physical and/or mental health conditions.^4 5^ Care provision often lacks integration and individualisation, with staff inexperienced in managing complexity.^6^ We previously showed that pregnant women with MLTC often feel responsible for facilitating communication between care providers, while also feeling patronised and disappointed that their expertise is under-recognised.^7^

National Institute for Health and Care Excellence recommends that pregnant women with MLTC are included in the development and review of their individualised care plan and are cared for by a multidisciplinary team led by a named healthcare professional.^8^ The Royal College of Obstetricians and Gynaecologists recommends enhanced postnatal care for such women, and that maternity networks should develop care pathways for women with medical conditions.^9^ In England, Maternal Medicine Networks have been established to provide clinical leadership and co-ordinate specialist multidisciplinary care for women with medical complexity, including MLTC.^10^

Despite existing recommendations,^6 8 9^ much still needs to be done to improve maternity care pathways and outcomes for women with MLTC. Poor access to maternity and medical records across different locations leads to loss of information, anxiety, duplication and gaps in care.^7^ Women describe mixed experiences regarding care for their different identities: as a pregnant person, a new mother and as someone living with MLTC. Postnatally, women and professionals recognise a downgrade in care, with lack of access to pre-pregnancy care teams leaving women to self-manage their (often worsened) conditions as a new mother.

One strategy to improve care is the development and implementation of a care bundle. Care bundles generally consist of three to five key evidence-based interventions for one area of care.^11^ The bundle elements, when performed collectively and reliably, should improve patient outcomes. Previously, successful care bundles have been implemented in maternity care, with the aim of reducing obstetric anal sphincter injury ^12^ and perinatal mortality (Saving Babies’ Lives series).^13^

The aim of this study was to work with key interest holders to co-produce a care bundle for (pregnant) women with MLTC that could be pilot tested and implemented in UK maternity services. It builds on our previous qualitative study exploring the care experiences of pregnant women with MLTC. This study is part of the wider MuM-PreDiCT (Multimorbidity and Pregnancy: Determinants, Clusters, Consequences and Trajectories) consortium (https://www.mumpredict.org/) working to characterise and understand the predictors, determinants, and consequences of MLTC in pregnant women.

## Methods

This study followed a three-step process: (1) a consolidated list of key components of care for women with MLTC during pregnancy was created through secondary analysis of qualitative data collected through semi-structured interviews with 57 women and 51 healthcare professionals; (2) the list of care components was explored during four co-production workshops; and (3) synthesis of findings from (1) and (2) to develop a maternity care bundle of 4–5 key care components for pregnant women with MLTC. Women with lived experience of MLTC in pregnancy were involved with the qualitative data analysis and contributed to all workshop discussions.

### Qualitative interview study

The qualitative interview (using semi-structured interviews and thematic analysis) study explored the experience and care of pregnant women with MLTC. The interview study methodology is fully described in Hanley *et al*.^7^ Ethical approval was granted by the NHS Wales Research Ethics Committee 7 (reference: Lumivero/WA/0021). All participants received study information and provided written informed consent before interviews, including consent for audio recording and anonymised quotations. Participants were eligible if they were living with two or more pre-existing long-term health conditions and were at least 28 weeks pregnant or had given birth within the previous 2 years. Women were recruited from across the UK via NHS sites, social media, third sector and professional organisations and the MuM-PreDiCT website. Healthcare professionals with experience of caring for women with MLTC during pregnancy were also included. Purposive sampling was used to ensure diversity in case mix, service context and participant characteristics. Interviews were conducted between March 2022 and May 2023 either online, by telephone or in person. Throughout this qualitative study, members of the research team reflected explicitly on their disciplinary backgrounds, clinical roles and personal experiences, and how these may have influenced data collection, analysis and interpretation. Team members were encouraged to challenge each other’s assumptions and interpretations during analysis discussions to enhance reflexivity and rigour.

Of the 57 women, the majority resided in England (n=37) and in urban settings (n=43), were 26 to 39 years old (n=51) and had diverse ethnic backgrounds (White British/Welsh/Scottish/Irish ethnicity, n=39; Mixed, n=3; Pakistani, n=3; Black British, n=2; plus, representation from 10 other ethnicities). Women were living with a range of mental, neurodevelopmental and physical health conditions. 24 women were living with two long-term conditions, and 23 women were living with three or more conditions. All women were between 28 weeks pregnant and/or up to 2 years postpartum.

Of the 51 professionals, 39 worked in England and roles were extremely varied. They included midwives working in hospital and community settings, and in specialist midwifery roles, doctors working in maternity services (eg, obstetricians, fetal and maternal medicine consultants, consultant anaesthetists, obstetric physicians and a neonatologist), other secondary care professionals (eg, psychiatrists, a haematologist, a diabetes consultant, a consultant neurologist and an advanced nurse practitioner in epilepsy) and community/primary care professionals (eg, general practitioners, a health visitor and infant feeding lead and a consultant in public health).

### Secondary data analysis

To identify care components to explore in coproduction workshops, secondary in-depth inductive thematic analysis of relevant coded data from the qualitative interview study was completed.^14^ Data labelled in earlier rounds of coding under ‘what worked well’, ‘what did not work well’ and ‘future recommendations to improve care’ were explored, and additional codes were developed and applied to potential distinct care components, which were then grouped into themes. The study team included individuals with academic and clinical expertise and lived experience of MLTC in pregnancy. Throughout the process, the research team considered their positionality, specifically their disciplinary and clinical backgrounds, personal experiences and their influence on interpretation. Following the first round of inductive coding, SH created a list of 21 care components. MB, ZV and BT reviewed coded data for any additional potential components and following team discussions and comparison of findings with existing literature, four additional components were added (involvement of the GP, established communication channels between smaller and larger sites, peer support and signposting to resources and support). Component names and descriptions were refined and agreed by the multidisciplinary study team, including our Patient and Public Involvement (PPI) group, to produce a long list of potential care bundle components to explore in the workshops.

### Co-production workshops

**Figure 1:**
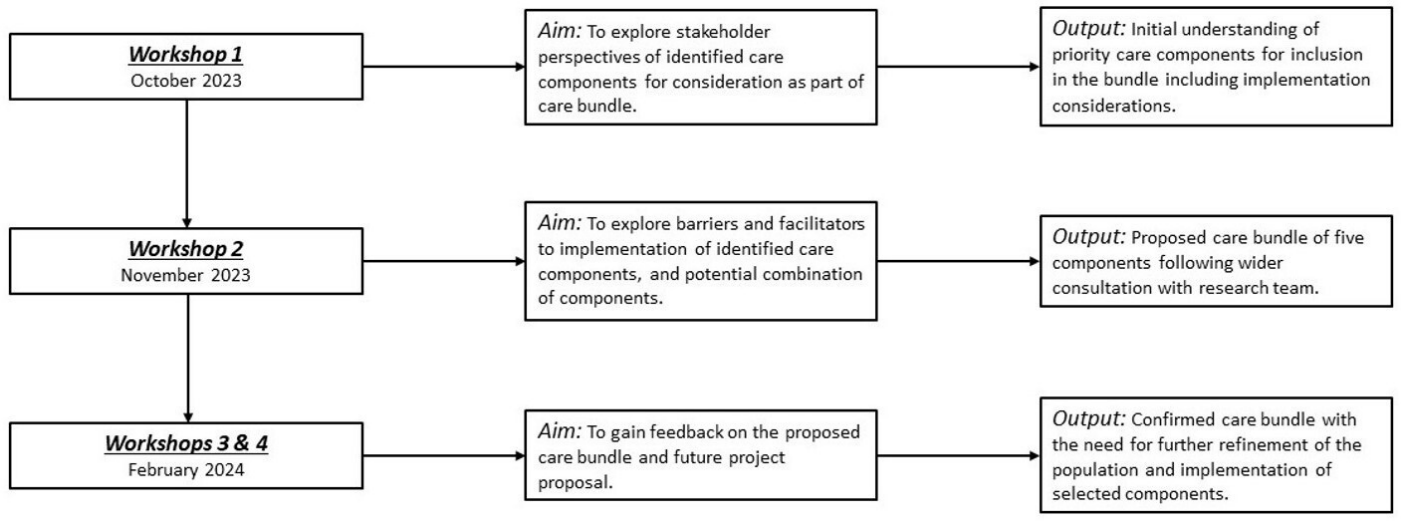
A summary of workshop aims and outputs.

Four online co-production workshops were conducted between October 2023 and February 2024 with 20–30 key interest holders in each (described in table 1). The workshops were designed to involve participants in refining the list of care components and care bundle development, specifically prioritisation of care components (workshop 1), identifying potential enablers and barriers to implementation of each component (workshop 2) and to provide feedback on the proposed care bundle and future research project proposal (workshops 3 and 4). Figure 1 summarises workshop aims and outputs. Workshop invitations were shared through community groups, voluntary organisations and charities, with interview study participants and the study team’s personal and clinical networks, and word of mouth. Healthcare professional invitations were focused on the key groups involved in commissioning, planning or delivering the care of women with MLTC before, during and after pregnancy. A core group of individuals was invited to all four workshops, supplemented by focused invitations guided by individual workshop aims. PPI group representatives and women who took part in the interviews^7^ attended each workshop. Women who attended the workshops were living with between two and six long-term conditions (physical conditions only=4 women, combination of physical+mental conditions=5 women).

**Table 1:** Co-production workshop participants

Interest holder group	Total across all workshops*	Workshop 1	Workshop 2	Workshop 3	Workshop 4
Total participants		20	27	15	18
Women and women’s organisations					
Women with MLTC who had used UK maternity services	9	8	8	5	4
Representative of National Maternity Voices	1	1	1		1
Representative of Maternity Voices Partnership	1		1	1	
Midwives					
Head of Midwifery Hospital Maternity Unit	4		3		1
Community midwife	1			1	
Hospital midwife	1			1	
Maternal Medicine Network Midwifery Lead	2	2	2		1
Midwifery manager	1			1	
Specialist midwife—public protection	1	1			
Specialist midwife—public health	1	1			
Senior charge midwife	1				1
Midwifery advisor for UK government	1		1		1
Midwife from NHS national leadership team	1		1	1	
Doctors					
Consultant obstetrician	7	3	3	1	3
General practitioner (family physician)	7	2	2	1	3
Consultant obstetric physician	2	1	1		1
Consultant neonatologist	1	1	1	1	
Maternal medicine consultant	1		1		
Consultant in medical specialty (specialty redacted)	1		1	1	
NHS hospital medical director†	1			1	
Specialist clinical pharmacist (hospital-based)	1				1
NHS hospital trust chief executive	1			1	
Representative of UKTIS	1				1
Regional maternal medicine network project manager	1	1	1	1	

* Some individuals attended multiple workshops.

† One individual had a split role as an obstetrician and medical director.

MLTC, multiple long-term health conditions; NHS, National Health Service; UKTIS, UK Teratology Information Service.

A list of potential care components was shared in advance of the workshops and all workshops lasted 2 hours. An overview of the MuM-PreDiCT project, including key findings from the qualitative interview work, was provided in each workshop. This was followed by two films prepared by our PPI coordinator, based on vignettes developed from the qualitative data delivered by women with lived experience. Each workshop involved full group and breakout discussions relevant to the aim of each workshop. All breakout discussions included a mix of service users and providers. Topic guides were developed to support all breakout discussions where two members of the research team were allocated to each breakout group—one to facilitate the conversations and one to record notes. At workshop 1, four breakout groups discussed their views and measurement of the different components, any new or missing components, important or priority components, the combination of any components and the implementation and resource requirements. At the second workshop, discussions around implementation continued where, in four breakout groups, participants provided feedback on how to implement each of the components and factors that may help or hinder implementation. At workshops 3 and 4, two breakout discussions were facilitated whereby, from a provider perspective, GPs and maternity staff provided input on the postnatal handover component and service leads, managers and representatives of national networks and organisations fed back on the care coordination component and the intended population for a future trial. At workshops 3 and 4, participants were also asked to vote in six polls related to the initial antenatal appointment processes, medication advice, continuity of care and access to clinical notes (online supplemental information 1). These questions were asked to understand local processes in different locations across the UK and to assess the feasibility of implementing care components. Participants were invited to share any further thoughts with the study team following the conclusion of each workshop. Throughout each workshop, the study team actively reflected on the discussions, recording their own reflections of participants’ perspectives on the importance of each component and how they interacted with one another.

### Moving from workshops to care bundle

During all workshops, research team members took contemporaneous notes using templates aligned with the workshop aims. Post-workshop debrief sessions enabled reflection on the key discussion points. Combined workshop notes were iteratively interrogated by the clinical lead (MB), with input from the wider team, to develop a proposed care bundle. Each potential component was assessed based on interview and initial workshop findings according to the following criteria: relative priority; practicality/feasibility; how it complemented other potential components while being a distinct entity; the potential impact on outcomes; and whether it addressed any aspect of good care which was not already addressed by another proposed bundle component.

### Patient and public involvement

PPI was embedded throughout the study. The PPI group contributed to refining and naming care components identified from the secondary analysis of qualitative interviews, ensuring that women’s lived experiences of MLTC in pregnancy informed all stages of the research.

PPI members participated in all four co-production workshops (October 2023–February 2024) alongside healthcare professionals and researchers. They helped prioritise care components, identify implementation barriers and enablers, and review the proposed care bundle. Two short films based on vignettes from qualitative data, co-produced with women with lived experience, were used to stimulate discussion.

Women attending the workshops were living with two to six long-term conditions, providing critical insights that shaped the final care bundle. The research team, including PPI contributors, reflected throughout on how their backgrounds and experiences influenced interpretation and co-production.

## Results

### Arrival at the care bundle

Secondary qualitative interview analysis and research team discussions led to a series of 25 care components being identified to improve maternity care for women with MLTC and healthcare providers.

Workshop discussions of the 25 components confirmed the importance of these, the overlap in how each could be provided and highlighted feasibility issues related to the proposed care bundle components and the intended population. Feasibility issues included potential challenges in identifying pregnant women with MLTC, lack of consensus on what constitutes expert medication advice, challenges around information governance when aiming to share summaries of care and resource limitations when implementing continuity of midwifery care. An agreement was reached recognising that these challenges are regularly overcome in practice and thus would require addressing in individual units to implement solutions that align with local service arrangements.

Following the workshops and synthesis of findings by the research team, a care bundle of five components was created which was predicted to have maximum impact across the range of areas of unmet need in maternity care for women with MLTC. Supporting information 2 presents the 20 care components that were not included in the care final bundle but were considered important in enhancing care for pregnant women with MLTC, and to a varying extent were considered likely to be addressed by the proposed care bundle components. Online supplemental information 3 presents the five care components that were selected for inclusion in the care bundle.

The final care bundle includes (1) early and reliable medication advice with decision support during pregnancy; (2) jointly accessible ‘Goals of Care Summary’ which flags risks and potential ‘red flag’ or worrying symptoms and signs; (3) continuity of midwifery care (community or specialist midwife) throughout pregnancy and postnatal care; (4) named care coordinator who is or works with lead obstetrician (could be midwife, obstetrician, obstetric physician, administrative or other); and (5) formal postnatal handover of care from multidisciplinary care team to the GP and secondary care teams involved in the woman’s care at time of hospital discharge or within 7 days of a home birth.

Online supplemental information 2,3 detail how the challenges identified in the interviews were translated into the listed care components based on evidence from the primary qualitative data collection. The rationale for including or excluding each component, along with key delivery considerations, is also presented. The majority of excluded components were addressed by the included components. For example, by including the ‘named care coordinator who is or works with lead obstetrician’ component, this also contributes to the full or partial achievement of a number of other components including ‘multidisciplinary early care planning’, ‘specialist review to determine whether specialist or midwife-led care should be offered’, ‘involvement of a specialist midwife’, ‘open MDT communication channels involving the woman as an equal partner’ and ‘checking that women are involved in their care’. Further, the study team decided not to include any pre-pregnancy components in the care bundle, as addressing these would require a much broader approach involving change in service provision beyond maternity and would involve an extensive network of additional interest holders working for different organisations.

**Figure 2:**
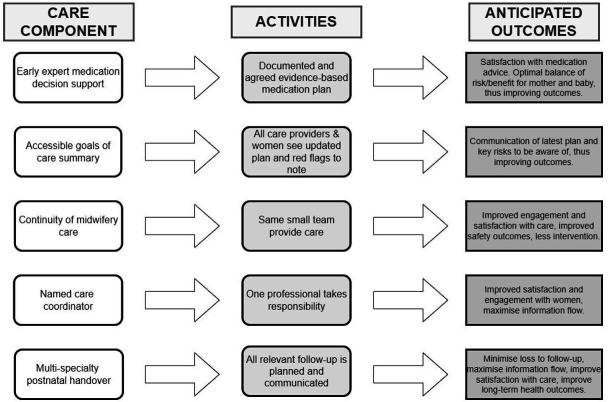
Flowchart of care bundle action.

The flowchart in figure 2 was created in collaboration with the research team to illustrate the anticipated outcomes from the successful implementation of each of the care components. Future multi-interest holder co-production is needed to refine the bundle and its activities in individual maternity units and to identify the most effective delivery strategies. Collectively, the care bundle should help to improve communication processes between professionals and with women, increase levels of care satisfaction, maximise information flow and, ultimately, improve the long-term management of women’s health conditions.

## Discussion

### Main findings

A maternity care bundle of five key care components for pregnant women with MLTC. These were provision of early and reliable medication advice and decision support to women; creation of a ‘goals of care summary’, accessible to women and the care team; provision of continuity of midwifery care (community or specialist midwife) throughout pregnancy and postnatal care; provision of a named care coordinator; and a formal postnatal handover of care from the multidisciplinary care team to the GP and secondary care team involving the woman.

### Strengths and limitations

The strengths and limitations of the qualitative interview study are described in Hanley *et al*.^7^ To our knowledge, the current study is the first exploring a care bundle for pregnant women with MLTC, offering a comprehensive analysis of the pregnancy pathway from preconception to postnatal care. The proposed care bundle, if implemented, may start to address areas of unmet need identified in our primary qualitative interview work and the wider literature, including reviewing the use of multiple medications in pregnancy and improving the postnatal handover process. By distilling our qualitative findings into an actionable care bundle, this study forms the foundation for a trial to evaluate whether the care bundle would improve outcomes for pregnant women with MLTC.

The study is underpinned by extensive qualitative work, providing valuable insights into areas of maternity care requiring improvement but grounded in the reality of challenges in implementing change in the current healthcare landscape. The four workshops, each focused on a specific question, facilitated the involvement of diverse interest holders, including women, practitioners, researchers and policymakers, fostering collaboration and shared decision-making. Involving these interest holders enabled identification of care bundle components which were deemed most important to women and providers of care, and also explored real-world considerations for implementation. The study team also conducted a rigorous evaluation of each potential component, by assessing its priority, feasibility, complementarity, potential impact on outcomes and ability to address elements of good care captured within other proposed components.

While virtual workshops facilitated attendance by busy individuals from across the UK, they have limitations. Compared with face-to-face events, online co-production can reduce interaction quality, participant engagement and development of personal connections and spontaneous interactions.^15^ Concerns about security, privacy and managing breakout discussions may have further impacted the effectiveness of the virtual workshops. The diverse nature of the interest holders in this work meant that some groups were less well-represented, for example, inclusion of multiple medical specialties outside of maternity. The observational nature of this study may limit the generalisability of the findings.

### Interpretation (in light of other evidence)

Implementation of a care bundle can enhance the quality and safety of healthcare through standardising care and adherence to best practices.^11^ A recent scoping review by Ryan *et al* encourages use of care bundles in maternity care and highlights the importance of considering all stages of pregnancy and birth to improve care.^16^ Established guidelines from the American College of Obstetricians and Gynaecologists and the NHS further support the use of structured care bundles in improving outcomes for women with MLTC, through enhanced care quality and consistency of care.^13 17^ A US-based study also highlighted the importance of multidisciplinary engagement, cultural assessments and evaluation of existing practices and context within a clinical setting to ensure care bundles are developed and implemented effectively.^18^ The proposed care bundle in the current work includes components that align with national guidelines and are evidence-based, yet results from our primary qualitative work demonstrated that no women experienced all these elements in practice (7). It is anticipated that the care bundle will provide structure and focus to the delivery of maternity care for women with MLTC, while channelling existing resources more effectively.

Earlier studies highlight that women face challenges in making decisions about medication use during pregnancy and require clearer information and improved communication with healthcare providers to make informed choices.^19^ Davies *et al*^20^ recommend a personalised written action plan and incorporating shared decision-making to involve patients in their medication choices at their preferred level of participation.^20^ Polypharmacy may be appropriate for pregnant women with MLTC if medicines are optimised and prescribed according to the best available evidence, while carefully weighing up the risks and benefits for both the mother and fetus.^21^ The findings from our qualitative interview study further highlight the importance of enhancing professional knowledge regarding the interaction between medications and pregnancy, while ensuring consistent communication across women’s care teams. Incorporating ‘red flag’ or worrying symptoms into care coordination plans, as part of the goals of care component within the current care bundle, has the potential to enhance both outcomes and care satisfaction, as previously evidenced in primary care settings.^22^

Our study adds to the existing literature base highlighting the value and impact of continuity of midwifery care, in improving maternal experiences.^23^ The need for further research into the impact of collaborative midwife continuity of care models for women with medical complexity has been identified,^24^ and it is crucial that existing issues with the implementation of such models are acknowledged and addressed.^25^

Findings from a prior systematic review examining maternity care coordination and its association with pregnancy outcomes showed modest improvements in birth weights among care coordination patients but the authors noted that future work was required to understand the effect on patient and provider satisfaction in antenatal care settings.^26^ Therefore, it is essential that our proposed care bundle incorporates both women’s and professionals’ input at every stage to maximise user and provider experience and satisfaction. The need for effective coordination of care for women with complex medical conditions, with involvement of multiple specialities and professionals, has been highlighted in recent UK and Ireland Confidential Enquiries into Maternal Deaths and Morbidity.^5^ In our earlier qualitative work and the workshop discussions, care coordinators were also deemed essential to provide continuity and oversight of care, connect providers and serve as a trusted, reliable support for pregnant women with MLTC.

Postnatal care is an essential component of the continuum of care for short-term and long-term maternal and child health.^27 28^ However, our earlier qualitative work highlighted that women with MLTC often ‘fall through the gaps’ postnatally, experience delays with re-engaging with pre-pregnancy specialist teams and, as a result, enter subsequent pregnancies with worsened health conditions.^7^ At the 6–8 week postnatal check, GPs also report difficulties in following up on women’s health conditions due to poor discharge summaries.^29^ This echoes the results of a previous review that identified challenges in achieving continuity of postnatal care, linked to discontinuities in handovers between healthcare professional groups, resulting in fragmented care.^30^ The inclusion of a formal postnatal handover of care in our proposed care bundle may therefore have the potential to improve outcomes for women with MLTC through the childbearing years and beyond.

Overall, it is crucial that care bundles are tailored to the specific needs of diverse ethnic and socioeconomic groups to address barriers to access and ensure equitable care for all women.^31^ Refining and integrating our proposed care bundle into routine clinical practice could significantly reduce disparities in maternal health outcomes, especially among under-served populations. However, careful design and thorough evaluation are crucial to ensure that existing inequalities are closed and not inadvertently exacerbated. Ensuring the scalability of care bundles across diverse organisations and populations is also crucial. Future research should focus on the evaluation of its effectiveness, implementation and cost-effectiveness in diverse clinical environments and populations. This will determine whether the care bundle can address the barriers to care for women with MLTC, including disjointed and siloed working, before widespread adoption can be recommended.^32^ Additionally, continuous professional development and training are crucial to ensuring healthcare providers are equipped to deliver optimal care to this group of women with MLTC. Ongoing education should prioritise clinical best practice, team-based working, cultural competence and women-centred care, with the goal of enhancing the effectiveness of interventions, such as the care bundle presented here, in improving maternal and neonatal outcomes.

## Conclusion

This work has coproduced a care bundle specifically for pregnant women with MLTC with the aim of improving outcomes. By focusing on evidence-based interventions which collectively address the aspects of care prioritised by interest holders, the care bundle should enhance communication among care providers and ensure personalised care which relieves pregnant women of the burden of responsibility for their care. Collaborating with women, clinicians, researchers and interest holders is essential to refine, implement and evaluate this care bundle. Further evaluation in practice is required to assess the feasibility of the care bundle and the impact on women’s outcomes, while ensuring it supports equitable and effective care across UK maternity services.

## Supplementary material

10.1136/bmjopen-2025-103366 online supplemental file 1

10.1136/bmjopen-2025-103366 online supplemental file 2

## Data Availability

No data are available.
