# Supplementary material for: Maternity care bundle for UK women with multiple long-term health conditions: coproduction workshops
Source: BMJ Open. 2026 Feb 6;16(2):e103366. doi: 10.1136/bmjopen-2025-103366 (PMC12887499; doi:10.1136/bmjopen-2025-103366)
Supplement: online supplemental file 1 [file bmjopen-16-2-s001.docx]

[Supporting Information 1: Workshop 3 & 4 Poll Questions 1](#_Toc476376608)

[Supporting Information 2: Summary of components of care from interviews with women and healthcare providers excluded from care bundle. 1](#_Toc511901149)

[Supporting information 3 Trustworthiness criteria 22](#_Toc1804641438)

**Supporting Information**

# Supporting Information 1: Workshop 3 & 4 Poll Questions

*Booking processes*

1. Staff only: How are women allocated to specific maternity care pathways? Is it based on…
2. Information from other professionals
3. Self-referral from woman
4. Other (free text in chat)
5. Staff only: Prior to the booking appointment with a maternity professional, how much information is shared on women’s health conditions?
6. Only information on referral form (from woman/professional)
7. Routine access to other records/information
8. Other (free text in chat)

*Medication advice*

1. Staff & women: Where do you access information on medications in pregnancy?
2. Pharmacists
3. UK Teratology Information Service (UKTIS)
4. Secondary care consultants (e.g., doctors caring for your health conditions)
5. Midwives
6. GPs
7. Internet
8. Other (free text in chat)

*Continuity of Care Practices*

1. Staff: Do you have midwifery continuity of care teams in your area? Women: Have you had experience of midwifery continuity of care in your pregnancies?
2. Yes
3. No
4. Are there any specific groups of women in your unit who receive midwifery continuity of care?

FREE TEXT

*Women’s access to portals & publishing of notes*

1. Staff & women: Does your unit currently use electronic maternity records?
2. Yes
3. No
4. Staff & women: Can women in your unit access any of their notes via an app or online portal?
5. Yes
6. No

# Supporting Information 2: Summary of components of care from interviews with women and healthcare providers excluded from care bundle.

| **Component of care** | **What existing problems may it ‘fix’? How might it improve care based off the findings from the background study?** | **Example quote from the background study** | **What will this look like?** | **How can this be delivered?** | **Why was the component excluded from the final bundle?** |
| --- | --- | --- | --- | --- | --- |
| *Pre-pregnancy* | | | | | |
| Multidisciplinary risk discussions and pregnancy planning | Women were generally satisfied with their preconception care when they received input from specialist and obstetric teams. However, there were instances where they received inconsistent advice on how pregnancy may impact on their conditions, including potential risks to the baby, as such many women conducted their own research. Staff described the importance of engaging in multidisciplinary pregnancy planning discussions to establish early contact and allow for quick referrals once the woman became pregnant. | “So, I was referred to see [name] at [hospital name]. She was brilliant, she said, “Yeah, that’s fine, we’ll write to [city] to say go ahead,” and she told me that once I was pregnant not to go through my GP, to make it all a slow process, to contact them directly to say I’m pregnant, and they’ll get me straight in on the obstetric systems for certain clinics, for the heart clinic specifically, but with epilepsy support. So, [city] went ahead.” (W46, Arrhythmogenic right ventricular cardiomyopathy, epilepsy, PCOS, endometriosis, anxiety, depression) | Formalised multidisciplinary preconception care pathways | Preconception planning with different services and people involved in a woman’s care outside of pregnancy | The study team believed that, at this stage, the pre-pregnancy work felt impractical as it requires a higher-level approach than a maternity perspective, with an enormous number of stakeholders involved. |
| Optimisation of health pre-conception including between pregnancies (linked to postnatal management) | Some women described preconception conversations to discuss any necessary changes to medication.  Staff also detailed the importance of preconception care to optimise health, discuss medications and link up the members of a woman’s care team to facilitate early planning. | “So, I think in a perfect world you would have these women with complex conditions who would be probably picked up by the GP in real life, and I think that you would have access for the GP to what I would probably, look I mean I’ll give it a name, I’ll call it like a complex care pathway, and that these women preconceptionally ideally, although not always possible, um, would be able to attend this clinic and number one in the clinic you could talk about contraception because most of these women have complex contraceptive needs as well. Number two you could talk about preconception advice, optimise everything and talk to them about what happens if they do get pregnant, and then following on from that the hope would be they can then continue in that service and that service I guess in a perfect world, practically speaking would need a co-ordinator. It would need to run like an MDT clinic. I think in real life you’d have to have some form of administrator to liaise with the various different specialities and make sure things come together.”  (S13, Sub-specialist trainee (year 5) obstetrics & gynaecology) | Working with the woman to, where possible, get her conditions in the most stable state before trying for a baby (e.g., blood sugar control in diabetes) | Preconception planning with different services and professionals involved in a woman’s care outside of pregnancy | The study team believed that, at this stage, the pre-pregnancy work felt impractical as it requires a higher-level approach than a maternity perspective, with an enormous number of stakeholders involved. |
| *Early pregnancy* | | | | | |
| Multidisciplinary early care planning | Women described inconsistencies in the advice received from different care team members and at different stages of their pregnancies. Women also described instances where decisions on care were made way too late (e.g., birth plans were not developed until late in the third trimester). Staff also shared the importance of early planning to ensure consistency and shared goals of care. | “It’s like from my first appointment, the first consultant I saw was like, “…under no circumstances are we doing a C-section on you unless it’s detrimental to you or your child because we’re not going to risk doing a C-section when your risk of bleeding is so high… but yet when I see another consultant they say, “Oh maybe the best option um as it is likely that you’re going to have a big baby is that we do a C-section”…. The Haematologist is like C-section is an absolute no, unless needs must. Um whereas the Obstetricians are the one that keep mentioning it…. And I genuinely think it’s because, because it’s not the same Obstetrician every time I go there, they don’t know me. They literally just look and see oh she’s this far gone but they don’t know what medication I’m on. I have to go over it every time I see one of them, what dosage of medication I’m on…. – It’s annoying because I feel like with the health conditions that I have, if it’s not possible to have the same person looking after my care, then they should at least read about me before I go in there, because we waste half of the appointment of me telling them things that they should already know.” (W40, Venous thromboembolic disease, raised BMI, 29 weeks pregnant) | Fast track referrals- booking appointment prior to 12 weeks | Pregnancy planning, with different services and professionals involved in a woman’s care | By already including allocation of a care coordinator, continuity of midwifery care and a goals of care summary it was felt that this component would be facilitated if the bundle is implemented effectively. |
| Specialist review to determine whether specialist or midwife-led care should be offered | Women described inconsistencies in care and level of specialist input between pregnancies and places. Staff detailed the importance of specialists providing holistic care. | “I moved up from [city] to [city] before I was pregnant, and so I haven’t really been… I was in a really good system down south for my proctitis, that’s where it was diagnosed, you know it was, the team were really good. But actually up here it seems that every… it’s obviously a really busy service, got a big waiting list, and I hadn’t really been in the system long enough before I got pregnant I think to get that care, and it was obviously Covid was going on, and everything was cancelled, so I hadn’t really spoken… I think I had maybe one or two telephone consultations with the gastro team, and I had to then let them know I was pregnant, and nobody really got back to me or took me up.” (W05, Type 1 diabetes, ulcerative proctitis, 11 months postpartum) | Early appointment to determine a woman’s care pathway- recognising the need for flexibility within ‘high risk’ and ‘low risk’ pathways | Consultant obstetrician | By already including allocation of a care coordinator and continuity of midwifery care it was felt that this component would be covered if the bundle is implemented effectively. |
| *Throughout pregnancy* | | | | | |
| Continuity of obstetric care | Women described many instances where they had to repeat themselves to different obstetricians, they were unable to build a relationship with anyone and they lacked holistic care. Some staff touched on obstetric continuity within, for example, a mixed midwifery and obstetric continuity of care model. | “Continuity of the care, that is not only just with the consultant, and also of community midwife, because community midwife provide equal number of or shared equal care with the consultant, so that oversees woman has got the direct contact with the community midwife than with us. So having that connection between the community midwife, consultant and the patient makes it much more easier for communication.” (S15, Maternal medicine consultant) | An obstetrician or small team of obstetricians to support personalised care, timely sharing of information, consistency in communication and development of a trusting relationship | Allocation of an obstetrician/small team of obstetricians for each woman | Midwifery continuity of care is already established in various Trusts so, at this stage, it is more feasible to focus on that rather than the introduction of a new continuity of obstetric care model which would have implications for obstetric staffing.  Gaps in care due to lack of continuity may also be partially addressed through use of the goals of care summary. |
| Involvement of a specialist midwife | Women spoke of HCPs lacking knowledge of their conditions and the effects on pregnancy which heightened women’s anxiety. Non-specialist midwives described feeling out of their depth when caring for women with complex health backgrounds. | “I have identified a skill gap because over time, and I’d, and I’d speak to colleagues, and I did a scoping review to say, “Listen what...” I sent an email out to them to say, “What exactly are your difficulties in terms of women with comorbidities?” and loads of people responded to say neurological, they don’t understand that, they don’t understand diabetes, they don’t understand epilepsy, they don’t understand, you know, the titration of drugs with preeclampsia because, because they have very little in University.” (S09, Midwife) | A midwife or small team of midwives with extra training (e.g., mental health, expertise in complex pregnancy) is part of a woman’s care team- providing support, information and care coordination (if appropriate) | Allocation of a specialist midwife | Not all Trusts and Boards have access to specialist midwives and not all health conditions necessarily require specialist midwifery care. By already including allocation of a care coordinator and continuity of midwifery care it was felt that where specialist midwifery care was warranted, it would be put in place via one of these routes. |
| Open MDT communication channels involving the woman as an equal partner | Women spoke of not being taken seriously/not listened to and decisions being made without her input. Some staff, especially midwives, believed that other members of a woman’s care team did not do a good enough job of engaging with women’s expertise and involving them throughout. | “And that’s before you get onto a service user trying to tell someone that what the situation is, and I have always been told by people that I am the best advocate of my health conditions, so then to not be listened and be gaslighted is like well I am not being funny or nothing, but I live with this every single day and they don’t.” (W16, Ehlers-Danlos syndrome, spondylolisthesis, severe asthma, functional neurological disorder, PTSD, 11 months postpartum) | Recognising the woman as an expert in her conditions and involving her in all care decisions. | All care team members with the woman | It is thought that this component will be largely addressed by the inclusion of other components in the bundle, including a care coordinator, a goals of care summary and a postnatal handover of care from the MDT. |
| Effective communication between professionals and with woman | Women described communication issues between HCPs such that care decisions were made extremely late, creating anxiety. Both women and HCPs highlighted challenges when care was delivered across multiple sites. | “So the advice from the hospital where I was going to give birth was very general to all pregnant ladies, so then I was like well I’m not happy, I want to communicate with my other specialists, just to make sure that I am doing the right thing by me and the baby. So yeah it was difficult having to always… every time I had any appointment I’d have to let the other team know, and it just didn’t feel like they were communicating on my behalf, and I had to feel like no I need to make sure.” (W37, Crohn’s disease, recurrent blood clots, 8 months postpartum) | Establishing agreed approaches to how information is shared between everyone (what, when, how). May include training and the use of online platforms to facilitate MDT team meetings. | All care team members including the woman | It is thought that this component will be largely addressed by the inclusion of other components in the bundle, including a care coordinator, a goals of care summary and a postnatal handover of care from the MDT. |
| Established communication channels between smaller and larger health facilities | Women described that HCPs lacked confidence in providing care at smaller sites where specialist knowledge was not available. HCPs experienced difficulties in accessing advice from those specialists at tertiary sites. | “Sometimes it’s a condition that the obstetrician is maybe not familiar with and doesn’t appreciate is relevant. Sometimes as I say it’s the smaller [hospital name]… not that I’m dissing [hospital name], but because they obviously don’t have the numbers and they don’t see us, I don’t know the obstetricians in [hospital name] because I don’t work with them. I know all the obstetricians here, and they know all my colleagues here, so we probably have a little bit of they sometimes get late referrals from [hospital name] possibly because their pathways aren’t working quite as well.” (S49, Consultant obstetric anaesthetist) | Establishing communication channels to allow for the delivery of timely advice (e.g., Maternal Medicine Networks in England) | All care team members including the woman | It is thought that this component will be addressed by the inclusion of a care coordinator and a goals of care summary. Aligns with existing service specifications for maternal medicine networks in England. |
| Open, transparent communication | Women described instances where care decisions were made without them, and they were not informed of potential events that may occur during and after the pregnancy (e.g., likelihood that the baby would need to be admitted to the neonatal unit). Staff described the importance of engaging with women at all stages of their pregnancies ensuring understanding and shared decision making. | “I certainly think one passing sentence I would like to say is never dismiss what a woman is saying. So, if a woman is crying or she’s gone to you because she feels so terrible, and she feels that something seriously is happening to her never ignore it. Women with complex needs it doesn’t necessarily just have to be them, but always think that this woman knows her condition much more than anyone else, and even if her suspicions or her concerns or her anxiety is unfounded she has anxiety about that, and it’s the clinician or the specialist’s responsibility to listen and act.” (S03, Epilepsy specialist midwife) | Establishing communication channels to allow for women to be involved at all stages of their pregnancy. | All care team members including the woman | This component will be covered within the bundle as it will be part of the care coordinator’s role to ensure open communication including the woman. It will also be facilitated by the regular updating of the goals of care summary. |
| Access to maternity records for all involved | Women spoke of having to share information and care updates with members of her team due to everyone not being able to view her health records (e.g., inside and outside of maternity). Staff also shared their experience of encountering barriers to accessing all required information due to the setup and access to digital notes. | “…the other issue that arises is that often a misconception on the obstetric side where there’s this idea that sometimes if somebody’s had a blood clot, we must induce their labour. And that’s not correct, and induction of labour leads to more complications. So, I think the communication, as long as people read the electronic notes is good, but when it’s, when it’s relying on a failure to read, then there’s a problem because there still leaves these kinds of archaic approaches.” (S19, Consultant haematologist) | Establishing ways for maternity records to be shared and accessed by all | All care team members including the woman | The inclusion of a goals of care summary will allow for all members of a woman’s care team to access key information and document changes throughout the pregnancy. Across the UK, NHS Trusts use different maternity paper based or electronic health records systems and it is not feasible to streamline this as part of the care bundle. |
| Involvement of GP | Women described instances during pregnancy where they required input from their GP, but the GP had no information on their antenatal care. Women and staff also described many issues postnatally where the GP was required to rely on the woman to share details of her maternity care and birth due to a lack of involvement during pregnancy. | “…If GPs are not looking after pregnant women every day…they automatically will lose confidence. So, you can’t ask GPs to spend a lot of time on educational training and resources, for stuff that they’re not going to do very often, because it just doesn’t work. Um, I suppose I’ve got a bit of a bee in my bonnet really about the fact that every time there’s a problem, with nearly every condition, people say, “Well, we just need to train the GPs more, we just need to, you know, we just need to educate them.” And, actually, that’s not always the answer. GPs have to know an awful lot, about an awful lot of stuff. And therefore, if they’re not going to be seeing people in pregnancy, what they really do need to know is the really important stuff, like, how to identify a pregnant woman who’s going into heart failure, like that’s an important thing to know.” (S10, General practitioner) | Including the GP in pregnancy care (e.g., ‘touchpoint’ appointments with GPs and/or the GP may be a member of the multidisciplinary team) | GP | This component will be addressed by the bundle as a) it will be part of the care coordinator’s role to ensure communication with the GP where relevant, and b) the GP will be provided with a formal handover of care following the pregnancy |
| Hospital admission plan | Women shared experiences of being admitted to hospital for the birth and unscheduled care reasons and not knowing the staff who were providing care. Staff had often not read or did not have access to women’s notes/they were unaware of women’s backgrounds which led to mismanagement of women’s conditions and poor birth experiences. | “But most of my problems came when I had to go into a maternity assessment because of reduced movements or other issues affecting pregnancy that the continuity of carer team couldn’t see, and in that situation they can’t always be there with you, and when they weren’t there with me it was very difficult, and the couple of times that my named midwife did manage to be there with me, even she turned round and said that it was ridiculous that they just didn’t seem to understand where I was coming from when I was trying to tell them that I was having to manually palpate movements, or that I have no feeling from navel down and I can’t wait bear and transfers etc.” (W16, Ehlers-Danlos syndrome, spondylolisthesis, severe asthma, functional neurological disorder, PTSD, 11 months postpartum) | Creation of an admission plan agreed early in advance and clearly recorded for women planning hospital birth and for unscheduled care admissions. | All care team members including the woman | Critical components of this will be included in the summary of care goals.  This component will also be addressed as it will be part of the care coordinator’s role to ensure a clear plan is documented for admission where relevant. |
| Birth plan clearly recorded and agreed | Women shared stories of birth plans being created late without input or agreement from all members of her team and without a multidisciplinary understanding of the potential impacts women’s health conditions. Staff detailed the importance of multidisciplinary planning (including the woman) for different eventualities dependent on the nature of women’s health conditions. | “One of the other conditions was the fear of giving birth naturally, so I discussed with my midwife that was quite close with me, and she’s the one that I had most of my appointments with, and I discussed with her my concerns and how I would prefer a C-section over a vaginal delivery. For me, I needed the consent form signed quite early on in pregnancy to say that they have given me permission, because a couple of doctors came in and this is probably four or five months into pregnancy and they have come in and said that, “You can’t actually have a C-section unless we sign it off and give you permission to have it.” And when I went back with that information to my midwife she said, “That’s not actually true, because it’s entirely up to you, it is your first pregnancy, it’s your body, and you have got a valid reason to go or push for the C-section.” So that was quite challenging because every time I’m getting told one thing, another person says something else, and that form wasn’t getting signed off. It needs to be signed by two doctors I believe to book in a planned C-section, and it just wasn’t getting done… they didn’t actually sign it off until I would say probably week 35/36, so I was very, very close to giving birth, and by then the fears and the anxiety had just crept up. It had multiplied by God knows how many, it was just really bad.” (W30, Anxiety, panic disorder, 11 months postpartum) | Initiating early multidisciplinary conversations to create a clear birth plan | All care team members including the woman | This component will be covered within the bundle as the care coordinator will work with the lead obstetrician and other members of the woman’s care team to ensure that conversations regarding birth plans happen early and in consultation with all key members including the woman. The goals of care summary will signpost to the birth plan. |
| Checking that women are involved in their care | Women regularly mentioned that they felt care discussions and decisions took place without them. Some staff members, especially midwives, shared the importance of engaging with women’s expertise in their conditions to facilitate women-led care. | “Never undermining the woman’s knowledge as well, because you need to do that assessment, because the woman may be extremely knowledgeable about her own condition, and whatever you must do you must never patronise that knowledge, and it's about building up on that knowledge wherever the woman is starting from.” (S03, Epilepsy specialist midwife) | Regular check ins with the woman at all stages of pregnancy and allowing her the opportunity to input into future care plans | All care team members including the woman | The inclusion of continuity of midwifery care and a care coordinator within the bundle will cover this component whereby the woman’s midwife/small team of midwives and/or care coordinator will check in with the woman at various points to check if she feels involved. |
| Peer support | Women hugely valued support from other women also living with multiple health conditions but often did not know where to access support. | “It would be really beneficial to give people support groups where there’s other people that are similar to them, because I reached out to a charity while I was pregnant, Pregnancy Sickness Support, and that literally saved me through my pregnancy. They allocate you a peer who has your number and you have theirs, and you just speak whenever you want to, it can be every day, it can be once a week, it can be however often. They are basically just like your little support person that just helps you through it, you can talk to them about normal life, you can talk to them about your pregnancy. It’s literally just like texting a friend, and I still speak to my woman now, and that’s a charity, that’s not through the NHS. I think the NHS would really benefit, I know they are really short for everything at the moment, but women really, really benefit from that.” (W15, Functional neurological disorder, anxiety, depression, 4 months postpartum) | Alerting women to peer support groups that may be of interest. | Midwives? | Peer support was not under the jurisdiction of the NHS and thus it did not seem feasible to influence it in a trial, especially as the nature and quality of peer support available could not be controlled by researchers. |
| Signposting to resources and support | Women shared many accounts of having to complete their own research to understand the interaction of their conditions with pregnancy. | “So even if they just give a leaflet with reliable sources of information, so I know somebody who has got one of these Ehlers-Danlos syndromes, and like there’s a specific website for that, so they can say you can go on this website and this will give you good information, because there’s a lot of sxxx information out there on the internet.” (W09, Endometriosis, hypothyroidism, sciatica, depression, anxiety, 21 months postpartum) | Direct women to evidence-based educational resources and support groups. | All care team members | The inclusion of continuity of midwifery care will allow for women to build relationships with their midwife/midwife team who will be able to signpost women to relevant support groups and educational resources. |
| *Postnatal care and planning for future pregnancies* | | | | | |
| Postnatal care planning | Women and staff both described issues with women’s transition back into pre-pregnancy care with long delays in obtaining appointments to discuss conditions that may have been exacerbated during pregnancy, and associated lack of guidance from professionals on any required medication changes. As such, women were often left to manage their worsened health conditions whilst also being a new mother. | “I think the postnatal care of these ladies is poor. Let me be honest, you know. And I think the reason for that is number one where, where any pathway exists it’s very antenatally focussed. Number two, is that often postnatal women get seen by the most junior members of the team, and that’s not good, more for the women I suppose. It’s good for their training but not for the woman and what that means in real life is that we’ve got no clear postnatal plan on the management of their complex conditions, they’re being seen by the most junior person who doesn’t necessarily have the, the coping mechanisms that, that other people have developed over time, and so what tends to happen is they go ‘oh well you’re fine now just leave and then you know sort it out with your GP later’. (S13, Sub-specialist trainee (year 5) obstetrics & gynaecology). | Appointment with woman and GP or multidisciplinary team or obstetrician (separate to appointment for baby) to discuss current healthcare needs, and plans for ongoing care and follow-up, including any changes to medication. | Multidisciplinary care team | The inclusion of a formal postnatal handover in the care bundle will include details of how women will transition back into pre-pregnancy care. |
| Detailed discharge summary for GP | Related to ‘involvement of the GP’, GPs shared details of discharge summaries that were often brief and lacked key information, as such they had to rely on the women’s accounts. Other professionals shared their sympathy for GPs who were expected to ‘pick up the pieces’ after pregnancy. | “Then postnatally all those precious antenatal notes are inaccessible to us. So, I don’t know when her labetalol was increased and I’ve got no way of finding that out, because the birth discharge summary will just say had a live baby it weighed four kilos, had a second degree tear. I will get told how long the second and third stage of labour was, which is almost never going to be of any relevance to me, but I won’t get told necessarily the doses of antihypertensives that she’s on, or a plan for weaning her metformin, not always, occasionally you will. So, it’s about what’s in the discharge summary, but it’s also about shared patient records contemporaneously as well.” (S18, General practitioner) | Improve the quality of and detail included in discharge summaries (e.g., create a template within which key information must be reported) | Members of a woman’s discharge team | Women with MLTC often have multiple care providers, many of which are in secondary care. Thus, it was not felt that a detailed discharge summary for the GP alone was justified, but instead that a more comprehensive postnatal handover was required to ensure the whole care team were engaged in the process. |
| Contraception | Staff shared their views on the importance of robust post-delivery contraception discussions with women with MLTC. | “So, patients with complex cardiac disease or any multi-morbidity they need a very robust plan for contraception after delivery, and you can even give them an implant on the postnatal ward, which is often the best thing if the woman is agreeable to that.” (S07, Consultant in fetal and maternal medicine) | Initiate discussions around contraceptive needs and wishes at an appropriate time following delivery. | Members involved in postnatal care | Contraception discussions and decisions will be included as part of the postnatal handover. |

*KEY:* Components coloured in light grey were added to the list following the first round of sub-coding.

**Supporting Information 3:** Summary of components of care from interviews with women and healthcare providers included in the care bundle.

| **Care bundle component** | **Rationale for inclusion** | **What will delivery look like?** | **Evidence from background study (staff)** | **Evidence from background study (women)** |
| --- | --- | --- | --- | --- |
| Early and reliable medication advice with decision support during pregnancy | This component was included as it was strongly emphasised by women and staff in the background study and workshop discussions. Staff shared difficulties in reversing medication advice and women described a lack of shared decision making around continuation or not of medications which often led to inconsistent advice and women having to complete their own research to make informed decisions. | Early documented conversations about medication with the lead obstetrician or obstetric physician which adequately addresses maternal/fetal risk balance. Content of medication advice should come from one or more of pharmacist advice, authoritative publications including UK Teratology Information Service, and expert knowledge. | “It feels very hard to undo the conversation where someone is told it's dangerous, because somebody has planted the seed of doubt than it is to change… Someone said “yes, they can do it”, and then you say, “Maybe on balance you shouldn't.” I don't know, it's much harder to undo. As soon as you have given the woman a lack of confidence that the medication is safe for her baby it's really difficult.” (*S23, Consultant obstetrician physician*) | “Well nobody really told me the risks either way, they just said, “Stop it, just stop,” and then like I said the other guy said, “Oh well it’s up to you.” but nobody really explained to me what would happen, what could happen, and again I wish I had asked, but at that point in my life I trusted these people to tell me everything, I don’t now, I really don’t.” (*W10, endometriosis, PCOS anaemia, 4 months postpartum*) |
| Jointly accessible ‘Goals of Care Summary’ which flags risks and potential red flag symptoms and signs | This component was included as it was judged to be high priority and feasible. In the background study, women shared many experiences where professionals were not aware of changes to their care plans or had not read their notes/been able to access updates on online systems. Staff described similar experiences whereby updates to care had not been shared or discussed as a team and the information could not be accessed. Staff shared examples of MDT arrangements whereby individualised care plans were developed which, crucially, included early postnatal and inter-pregnancy planning. It was also thought that this component would contribute to the partial achievement of other care components. | On meeting the woman for the first time, the lead clinician will develop an accessible summary of care, including conditions that have the potential to affect or be affected by the pregnancy, a provisional plan for their management and a list of red flags for care providers and the woman to look out for, why these are important and what action to take.  Later changes to management, for example, medication, and signposting to key entries in the notes will be included. This will be stored in an accessible place e.g. in a summary section of an electronic maternity record and copied into a non-maternity record for the wider care teams to see. | “The last one that I went to was the liver MDT… one of the consultants had been through and collated all of the liver patients, because they’re all seen in a joint clinic, and their care was discussed each one individually from both sides. So these were pre-pregnancy patients that had been seen or currently pregnant, or postnatal, so they were all included in this MDT, and it happens once a month. Any investigations that need to be organised are, and generally it would obviously be the liver team that would then follow them up postnatally, so making sure that there’s a clear plan for next pregnancy as well if that was planned.” (*S06, ST6 fetal and maternal medicine*) | “They have changed my hypertension medication and they have put me on… but again I don’t know what they have put me on, because I was taking so many pills. But I am sure they put me on something, but no one came and explained to me this is what you are taking for this.” (*W12, rheumatoid arthritis, hypertension, 20 months postpartum*) |
| Continuity of midwifery care (community or specialist midwife) throughout pregnancy and postnatal care | This component was included as it was strongly emphasised by women and staff in the background study and workshop discussions. Women described many instances where they had to repeat themselves to different midwives, they were unable to build any relationships and conflicting advice impacted on their levels of trust. Midwives mentioned examples of continuity of carer in current and/or past workplaces, but staffing issues impacted on the ability to deliver the model. It is supported by the existence of midwifery continuity of care models nationally (although not universal).  In the background study, staff detailed the importance of involving a specialist midwife in the care of women with MLTC to provide continuity and reassurance and woman also described the importance of continuity of midwifery care in preventing the need to repeat the same information at every appointment.  Inclusion will also make a major contribution to the partial achievement of various other care components. | Antenatal care and postnatal care provided by one or a small team of midwives to support personalised care, timely sharing of information, consistency in communication and development of a trusting relationship. This does not necessarily need to include intrapartum care in the context of the care bundle, as other aspects of the bundle should ensure that continuity of information sharing is optimised across the care journey. How this care will be provided (to women with different types of MLTC) should be agreed at site level, recognising that specialist midwives may do so for severe MLTC in tertiary centres, while community midwives may do so in smaller centres or when MLTC requires less obstetric input. | “[hospital name] have got an amazing service, and there’s a specialist midwife there who does an epilepsy clinic, and she’s got a lovely video clip, so if you get a chance you should watch the video clips that she had done, and that really what sings through is that it’s a relief for the patients to be able to have their epilepsy nurse and their neurologist in the room with the midwives so that everyone is… you don’t have to tell your story three times.” (*S35, Advanced nurse practitioner (epilepsy)*) | “But seeing a different midwife every time, and having an appointment that’s 17 minutes, and it feels like you’re in the door, out the door, it’s like it’s just really crappy, and they haven’t got time to read your notes, when you see the different one they ask you the same thing that you have already said that’s already written on your file, and it’s just like you know that they have been in clinic all day and that they are really tired, and they are just trying to shorten the appointment so that they have got time to write up their notes and see the next person, and I think continuity of care and being able to see the same midwife is so important, but it’s just not… I know other trusts do it, but the trust that I’m in it’s just not physically possible.” (*W13, dyspraxia, ADHD, 37 weeks pregnant*) |
| Named care coordinator who is or works with lead obstetrician (could be midwife or administrative or other) | This was included due to it being high priority and the existence of care coordinators in other health care settings (e.g., cancer, mental health, primary care) showing effectiveness. In the background study, women and staff described issues with women being bounced between different teams, having to repeat themselves at appointments and take on the care coordinator role. In a few cases, both women and staff detailed examples of dedicated care coordinators (e.g., specialist midwives and obstetric physicians) in their local Trusts. Staff shared that “co-ordination of care is quite challenging, because there isn’t a one person to whom it comes to” and the lack of existence of these roles within maternity was recognised.  It was also considered likely to contribute to the partial achievement of various other care components. | A named person to provide oversight and joined up care across different specialists and services- facilitating ongoing conversations and updating the team as appropriate. This would be the key point of contact for the woman. This could be a continuity of care MW, a specialist MW, a lead obstetrician or obstetric physician, depending on setting and conditions involved. The coordinator would take responsibility for ensuring the bundle components were implemented by relevant members of the team. They could facilitate information sharing where gaps in access to information systems exist e.g. if a GP cannot access maternity notes or community midwives cannot access secondary care investigation results. | “If there’s a cancer MDT it’s not the consultants who are responsible for having to generate the lists of patients all the time, there will be a specialist either administrator or specialist nurse who has the lists of patients and then will bring it to the meeting, will make sure these patients are being followed up, and then we’ll talk about them, and the consultants will be then planning the management of that patient and communicate the management to the patient and delivering the management to the patient. In the pregnancy teams it’s us having to do all of that stuff as well, which is problematic, because you’re not the best placed person to do it.” (*S07, Consultant in fetal and maternal medicine*) | “Because no one communicated with each other, if I had a gynaecology or whatever, an appointment for the baby, like an ultrasound scan or what have you, then I’d have to tell my consultant in [city], and the one in [city], and my haematologist, and they didn’t really communicate. So, I would… me because I’ve had long term health conditions for a while, always go to appointments with letters, and questions from other departments, because I know that some things get lost, or they don’t always go to. So, it’s quite stressful having to make sure that you’re on the ball and everyone knows the right thing, and everyone knows the right up to date thing that’s going on.” (*W37, Crohn’s disease, blood clots, 8 months postpartum*) |
| Formal postnatal handover of care from multidisciplinary care team to the GP and secondary care teams involved in the woman’s care at time of hospital discharge or within seven days of a home birth | This was a high priority component considered feasible and likely to attend to many of the postnatal care and handover issues described in the background study, as well as contribute to pre-pregnancy planning for any future pregnancies. In the background study, both women and staff detailed breakdowns in care and communication between pregnancy teams and ‘usual’ care providers. Additionally, staff described their concerns at the potential for women to ‘fall through the gaps’ due to a lack of formalised postnatal care pathways. National efforts are also concentrated on improving postnatal handovers. | Formal and detailed handover of care from pregnancy care team with agreed and documented follow-up plans, including how the woman will transition back into pre-pregnancy care (who does she need to see and when, who needs to know what information etc.) with the aim of preventing a worsening of a woman’s health conditions and ensuring holistic care. This will be drafted by the MDT, including the woman, during the antenatal period and finalised prior to discharge from hospital (or from community midwifery care after a homebirth) with updated information from the peripartum period. The care coordinator may inform the MDT of what happened during the woman’s birth to inform the discharge plan. | “Have you got the right follow-up for this woman? Are they going back to their specialist physician? If they weren’t under a specialist physician before are they going to get the right follow-up, have they just had pre-eclampsia, are they going to get their uro check with their GP in six weeks, is that actually going to happen, or are they going to fall by the wayside and then come back two years later pregnant with more medical problems that haven’t been sorted?” (*S17, Consultant obstetrician*) | “I think that then really muddled the care of my blood pressure after the second pregnancy, because I think the GPs didn’t really want to make a decision because I had cardiology input, and the cardiologists had got their back up a bit because I was under the renal team, and so the cardiology ended up I had an appointment with… a phone call appointment with them, and I mentioned something about oh I don’t know what medication is going to be best for my… to protect my kidneys going forward, and I meant it as a throwaway comment, but it came back from the consultant as, “Oh you want to be under the care of the renal consultant, so we’re going to discharge you.” So, I ended up being discharged from the cardiologists with no… what a plan for follow-up from the renal team, but no appointment yet from the renal team, and then I think the GPs were like, “Well you’ve got this plan to see the renal team.” So, I think having almost too many teams involved made it a bit more complicated after my second pregnancy.” (*W47, Chronic kidney disease, hypertension, inflammatory bowel disease, idiopathic intracranial hypertension, 12 months postpartum*) |

# Supporting information 3: Trustworthiness criteria

| Trustworthiness criteria | Examples of strategies from our study |
| --- | --- |
| Credibility | Reflexive approach, multiple researchers and active reflection and diary-keeping, acknowledging personal biases  Sampling of interviews from diverse individuals and contexts. |
| Transferability | Clear description of the research context and participants in paper reporting the main findings.  Clearly articulated sampling process and criteria. |
| Dependability | Clear documentation of the procedures and decisions throughout the study and in reporting, according to standard criteria. |
| Confirmability | Involvement of diverse individuals in the research team, and in interpretation of the data, including people with lived experience.  Reflexive journalling by the researchers conducting and analysing the interviews. |
