# Supplementary material for: Maternity care bundle for UK women with multiple long-term health conditions: coproduction workshops
Source: BMJ Open. 2026 Feb 6;16(2):e103366. doi: 10.1136/bmjopen-2025-103366 (PMC12887499; doi:10.1136/bmjopen-2025-103366)
Supplement: Supplementary file 3 [file bmjopen-2025-103366.reviewer_comments.pdf]

PEER REVIEW HISTORY

BMJ Open publishes all reviews undertaken for accepted manuscripts. Reviewers are asked to complete a checklist review form and are provided with free text boxes to elaborate on their assessment. These free text comments are reproduced below.

ARTICLE DETAILS

Title (Provisional)

A maternity care bundle for UK women with multiple long-term health conditions: coproduction workshops

Authors

Hanley, Stephanie; McCann, Sharon; Singh, Megha; Lee, Siang Ing; Plachcinski, Rachel; Nirantharakumar, Krishnarajah; Black, Mairead; Locock, Louise; Taylor, Beck

---

VERSION 1 - REVIEW

**Reviewer**	**1**
**Name**	**Asaye, Mengstu**
**Affiliation**	**University of Gondar College of Medicine and Health Sciences, midwifery**
**Date**	**18-May-2025**
**COI**	**None**

Dear author, thank you so much to produce this really original and therapeutically essential scientific study. I read it thoroughly. It is an excellent work.

Tittle: A maternity care bundle for women with multiple long-term health conditions: co-production workshops

- ✓ Well written

My comments may provide some valuable insights for further improving your work.

Abstract:

- ✓ **Objective**

  The objective of this study is to co-produce a care bundle for women with multiple long-term health conditions (MLTC) "that could be implemented and tested" in UK maternity services. Comment: it would be nice to revise like …that could be "pilot tested and implemented" in UK maternity services. Because first it could be pilot

tested, then if it could be effective, it could be implemented……

- ✓ **Design**

  Online co-production workshops each attended by 20-30 key "interest holders". Dear author, the term" interest holders" could not include all of your study populations. The study populations were: [pregnancy with MLTC, healthcare professionals]??? and other interest holders. Better to use appropriate inclusive words. May be to use " study participants"

- ✓ **Conclusions**

  Line 47-48 "By focusing on evidence-based interventions," this phrase is not necessary.

Introduction

Well written

- ✓ Line 14: Please write it "NICE" in full. The same is true line 17 for " RCOG"
- ✓ Line 39: " The aim of this study was to work with key interest holders to co-produce a care bundle for women with MLTC that could be implemented and tested in UK maternity services".
- ✓ Dear author, It could be more attractive and sound, if you revise like this: The aim of this study was to co-produce a care bundle for [pregnant] women with MLTC that could be pilot tested and implemented in UK maternity services

Methods

Qualitative Interview Study

- ✓ Dear author, even if you put the citation (methodology) but it would be nice to mention the type of qualitative study design used.
- ✓ Under your socio-demographic characteristics part, you mentioned residency and ethnic backgrounds. In case their educational level, and gravidity level might had impact on you result. What is your justification on this regard?
- ✓ The same is true for health care professionals. Your study participants were different health care providers with different roles. What about their level of education? It is better to highlight it there.

Dear author, as it was a qualitative study

1. How the thursworthiness was kept? This is very important.
2. Were there any positionality or reflexivity statements about the entire process

of your qualitative study? if so please attach in the additional file.

Discussion

Main findings

- ✓ Dear author, please focus on your key findings rather than including your objectives. This is not necessary" The aim of this study was to co-produce a care bundle for women with MLTC that could be implemented and tested in UK maternity services. Secondary analysis of qualitative data identified 25 potential care components which were refined to……" I recommend to delete it.
- ✓ **Here are your key findings**: "A maternity care bundle of five key care components for pregnant women with MLTC. These were; provision of early and reliable medication advice and decision support to women; creation of a 'goals of care summary', accessible to women and the care team; provision of continuity of midwifery care (community or specialist midwife) throughout pregnancy and postnatal care; provision of a named care coordinator; and a formal postnatal handover of care from the multidisciplinary care team to the GP and secondary care team involving the woman."
- ✓ Your **interpretation** will be in line with this key findings.

Conclusion

The conclusion in the abstract and in the main document are quit vary. Please work on it.

Dear author, it will be nice to add "**implication statements**" with regard to your study findings

---

**Reviewer**	**2**
**Name**	**Homer, Caroline**
**Affiliation**	**Burnet Institute**
**Date**	**15-Sep-2025**
**COI**	**None**

Thank you for the opportunity to review this paper. The objective of this study was to co-produce a care bundle for women with multiple long-term health conditions (MLTC) that could be implemented and tested in UK maternity services.

There needs to be a clear definition of what are multiple long-term health conditions. There is an assumption that the reader knows.

The methodology was online co-production workshops each attended by 20-30 key interest holders. There was a secondary analysis of prior collected qualitative data.

While I like the 5 components of the care bundle, I am not convinced that they will solve the key concerns raised of siloed or disjointed care. A further study needs to occur to actually show that these make a difference.

Many of the stakeholders were the only one of their profession included. For example, one specialise medical consultant, one pharmacist, one medical director etc. This raises concerns about such a limited perspective from the different groups. I also could not see general or specialist physicians outside of maternal health care. It feels like the non-maternity workforce are even more important in this project.

The study seems very UK focused. What is the relevance or utility in other countries?

MLTC is an unusual abbreviation and one that I have never heard of before. For an international audience I would not use the abbreviation.

VERSION 1 - AUTHOR RESPONSE

Reviewer: 1

Mr. Mengstu Asaye, University of Gondar College of Medicine and Health Sciences, University of Gondar College of Medicine and Health Sciences
Comments to the Author:
Dear author, thank you so much to produce this really original and therapeutically essential scientific study. I read it thoroughly. It is an excellent work.
Tittle: A maternity care bundle for women with multiple long-term health conditions: co-production workshops
My comments may provide some valuable insights for further improving your work.
Abstract:

Objective
The objective of this study is to co-produce a care bundle for women with multiple long-term health conditions (MLTC) "that could be implemented and tested" in UK maternity services. Comment: it would be nice to revise like …that could be "pilot tested and implemented" in UK maternity services. Because first it could be pilot tested, then if it could be effective, it could be implemented……

This change has been made in the manuscript Page 2 lines 3-4

Design
Online co-production workshops each attended by 20-30 key "interest holders". Dear author, the

term” interest holders” could not include all of your study populations. The study populations were: [pregnancy with MLTC, healthcare professionals]??? and other interest holders. Better to use appropriate inclusive words. May be to use “ study participants”

We have selected the term 'interest holders' as a more inclusive term than the more traditional 'stakeholders', in line with recent developments in policy and research nomenclature (Akl EA, Khabsa J, Petkovic J, Magwood O, Lytvyn L, Motilall A, Campbell P, Todhunter-Brown A, Schünemann HJ, Welch V, Tugwell P, Concannon TW https://onlinelibrary.wiley.com/doi/10.1002/cesm.70007). "Interest-holders": A new term to replace "stakeholders" in the context of health research and policy. (Cochrane Evid Synth Methods. 2024 Oct 29;2(11):e70007. doi: 10.1002/cesm.70007. PMID: 40475280; PMCID: PMC11795957 https://onlinelibrary.wiley.com/doi/10.1002/cesm.70007) .

The coproduction workshop participants were collaborators rather than consented research participants, therefore 'study participants' would not accurately reflect their status.

Conclusions
Line 47-48 ”By focusing on evidence-based interventions,” this phrase is not necessary.

Introduction

The conclusion has been edited as below page 2 line 28-30

“This study coproduced an evidence-based care bundle for pregnant women with MLTC to enhance communication and ensure individualised care and support. Further collaborative work with women and professionals is required to refine, implement and evaluate its impact on outcomes.”

Line 14: Please write it “NICE” in full. The same is true line 17 for ” RCOG”

The terms have now been defined in the manuscript

Line 39: ” The aim of this study was to work with key interest holders to co-produce a care bundle for women with MLTC that could be implemented and tested in UK maternity services”.
Dear author, It could be more attractive and sound, if you revise like this: The aim of this study was to co-produce a care bundle for [pregnant] women with MLTC that could be pilot tested and implemented in UK maternity services

This has been edited in the manuscript page 4 line 29-34

Methods
Qualitative Interview Study
Dear author, even if you put the citation (methodology) but it would be nice to mention the type of qualitative study design used.

Below has been added to the manuscript page 5 line 11-13

The qualitative interview (using semi structured interviews and thematic analysis) study explored the experience and care of pregnant women with MLTC.

Under your socio-demographic characteristics part, you mentioned residency and ethnic

backgrounds. In case their educational level, and gravidity level might had impact on you result. What is your justification on this regard? The same is true for health care professionals. Your study participants were different health care providers with different roles. What about their level of education? It is better to highlight it there.

We have reviewed and made changes to clarify the roles of healthcare professionals and ensure the descriptions are clear for an international audience. We have also considered the relationship between role and level of education, and revised the table accordingly.

Regarding participants' educational level and gravidity, we acknowledge their potential influence on experiences; however, these factors did not emerge as major explanatory themes during analysis. We have clarified this point in the methods/discussion as appropriate.

Dear author, as it was a qualitative study
1. How the thursworthiness was kept? This is very important.

This has now been addressed in the revised manuscript. As this study is based on a secondary analysis of qualitative interview data, we have clarified that detailed methods to ensure trustworthiness are described in the primary study. A brief summary has been added to outline key strategies used to maintain rigour, including researcher triangulation, reflexive discussions within the multidisciplinary team, and Patient and Public Involvement (PPI) input throughout design, analysis, and interpretation. We have also incorporated the supplementary table (or integrated the key points within the strengths and limitations section) to further support transparency.

Table below has been added in the supplementary information 3 (supplementary file 1)

Trustworthiness criteria	*Examples of strategies from our study*
Credibility	*Reflexive approach, multiple researchers and active reflection and diary-keeping, acknowledging personal biases*<br>*Sampling of interviews from diverse individuals and contexts.*
Transferability	*Clear description of the research context and participants in paper reporting the main findings.*<br>*Clearly articulated sampling process and criteria.*
Dependability	*Clear documentation of the procedures and decisions throughout the study and in reporting, according to standard criteria.*
Confirmability	*Involvement of diverse individuals in the research team, and in interpretation of the data, including people with lived experience.*<br>*Reflexive journalling by the researchers conducting and analysing the interviews.*

2. Were there any positionality or reflexivity statements about the entire process of your qualitative study? if so please attach in the additional file.

We have now added a reflexivity statement to the manuscript, adapted from the primary paper.

Page 5 line 23-27.

Throughout this qualitative study, members of the research team reflected explicitly on their disciplinary backgrounds, clinical roles, and personal experiences, and how these may have influenced data collection, analysis, and interpretation. Team members were encouraged to challenge each other's assumptions and interpretations during analysis discussions to enhance reflexivity and rigour.

Discussion
Main findings

Dear author, please focus on your key findings rather than including your objectives. This is not necessary" The aim of this study was to co-produce a care bundle for women with MLTC that could be implemented and tested in UK maternity services. Secondary analysis of qualitative data identified 25 potential care components which were refined to……" I recommend to delete it.

Here are your key findings: "A maternity care bundle of five key care components for pregnant women with MLTC. These were; provision of early and reliable medication advice and decision support to women; creation of a 'goals of care summary', accessible to women and the care team; provision of continuity of midwifery care (community or specialist midwife) throughout pregnancy and postnatal care; provision of a named care coordinator; and a formal postnatal handover of care from the multidisciplinary care team to the GP and secondary care team involving the woman." Your interpretation will be in line with this key findings.

This has now been edited accordingly

Conclusion
The conclusion in the abstract and in the main document are quit vary. Please work on it.
Dear author, it will be nice to add "implication statements" with regard to your study findings
Good Luck

The conclusion in the abstract has now been edited.

Reviewer: 2
Prof. Caroline Homer, Burnet Institute, University of Technology Sydney
Comments to the Author:
Thank you for the opportunity to review this paper. The objective of this study was to co-produce a care bundle for women with multiple long-term health conditions (MLTC) that could be implemented and tested in UK maternity services.There needs to be a clear definition of what are multiple long-term health conditions. There is an assumption that the reader knows.
The methodology was online co-production workshops each attended by 20-30 key interest holders. There was a secondary analysis of prior collected qualitative data.

While I like the 5 components of the care bundle, I am not convinced that they will solve the key concerns raised of siloed or disjointed care. A further study needs to occur to actually show that these make a difference.

We have added text to emphasise this in the discussion section page 12, lines 15-18

We agree that while the five components of the care bundle are important, they may not fully address the broader issues of siloed or disjointed care. We have added a statement to the discussion to emphasise this point and acknowledge that further research will be needed to evaluate the effectiveness of these components in improving integrated care.

Many of the stakeholders were the only one of their profession included. For example, one specialise medical consultant, one pharmacist, one medical director etc. This raises concerns about such a limited perspective from the different groups. I also could not see general or specialist physicians outside of maternal health care. It feels like the non-maternity workforce are even more important in this project.

We have added text in the limitations

In the workshops it was important to strike a balance between different groups, and this included a physician from outside of maternity (table of participants has been clarified, with specialty redacted for confidentiality purposes). The work was underpinned by qualitative research which included physicians from haematology, neurology and endocrinology specialties. Further limitations have been added.

The study seems very UK focused. What is the relevance or utility in other countries?

While this study is UK-based, the findings have relevance globally. MLTC (multiple long-term conditions) is an emerging area of clinical practice in maternity care worldwide, as the prevalence of MLTC is increasing across high-, middle-, and low-income countries. Existing evidence highlights the importance of multidisciplinary and integrated care models, though there remains a significant knowledge gap regarding the most effective care approaches internationally (Zoe et al., 2024; DOI: 10.1016/j.wombi.2024.101645).

MLTC is an unusual abbreviation and one that I have never heard of before. For an international audience I would not use the abbreviation.

The term MLTC is increasingly used in international literature to describe complex chronic disease combinations in pregnancy. For example:

- *Krauth et al. (2023) examined latent class analysis-derived multimorbidity clusters and adverse health outcomes across three UK cohorts, highlighting implications for patient management.*
- *Khunti et al. (2023) reviewed diabetes and multiple long-term conditions as a global health challenge, emphasising the need for effective care models.*

We could note that the older term "multimorbidity" has been widely used but is sometimes seen as having negative connotations (Black, Br J Gen Pract), though adoption of MLTC in maternity care literature is increasing.

VERSION 2 - REVIEW

Reviewer 1

Name Asaye, Mengstu

Affiliation University of Gondar College of Medicine and Health Sciences, midwifery

Date 10-Dec-2025

COI

Dear Author,

thank you very much for well addressing my feedbacks.

Good Luck

Mengstu Asaye (PhD)
